# Muscle loss and GLP-1R agonists use

**DOI:** 10.1007/s00592-025-02611-2

**Published:** 2025-11-07

**Authors:** Giada Rossi, Loredana Bucciarelli, Chyrell-Lyn Mananguite, Matteo Giovarelli, Paolo Fiorina

**Affiliations:** 1 International Center for T1D, Pediatric Clinical Research Center Romeo ed Enrica Invernizzi, DIBIC, Università degli Studi di Milano, Milan, Italy; 2 Division of Endocrinology, ASST Fatebenefratelli Sacco, Milan, Italy; 3 Pio Albergo Trivulzio, Milan, Italy; 4 Internal Medicine Department, Clemenceau Hospital, Johns Hopkins International, Dubai, UAE; 5 Department of Biomedical and Clinical Sciences (DIBIC), Università degli Studi di Milano, Milan, Italy; 6 Nephrology Division, Boston Children’s Hospital, Harvard Medical School, 300 Longwood Ave. Enders Building, Boston, MA 02115 USA; 7 Brigham and Women’s Hospital, Transplantation Research Center, Harvard Medical School, Boston, MA USA

**Keywords:** Muscle loss, GLP-1RA, Prevention, Treatment, Diabetes mellitus, Obesity

## Abstract

Skeletal muscle wasting is a major yet often overlooked determinant of adverse outcomes in diabetes mellitus and obesity. Loss of muscle mass and strength not only impairs mobility and quality of life, but also worsens insulin resistance, accelerates cardiometabolic decline and increases mortality risk. The convergence of chronic inflammation, mitochondrial dysfunction and altered protein metabolism makes individuals with metabolic diseases particularly vulnerable to sarcopenia. Glucagon-like peptide-1 receptor agonists (GLP-1RAs) have transformed the therapeutic landscape of type 2 diabetes (T2D) and obesity by offering substantial weight loss and cardiometabolic protection. However, clinical trials and real-world evidence consistently show that weight reduction with GLP-1RAs is accompanied by decrease in lean body mass, raising concern in patients already predisposed to muscle wasting and underscoring the need for integrated management strategies. By including all English-language studies on muscle mass loss during GLP-1RA therapy in T2D and obesity from major scientific databases and clinical trial registries, this narrative review synthesizes the current knowledge on the epidemiology and mechanisms of muscle loss in diabetes and obesity, with a focus on the impact of GLP-1RAs therapy. It further examines preventive and therapeutic strategies to preserve muscle health during pharmacological weight loss, with the ultimate aim of providing clinicians and researchers with practical insights and future directions to maximize the benefits of GLP-1RAs while mitigating the risk of sarcopenia.

## Introduction

A progressive decline in skeletal muscle mass and strength, or sarcopenia, is increasingly recognized as a significant comorbidity in patients with type 2 diabetes mellitus (T2D) and obesity [1, 2]. Multiple pathophysiological mechanisms, including insulin resistance, mitochondrial dysfunction, systemic inflammation, impaired anabolic signaling and reduced physical activity, contribute to accelerated muscle loss in these conditions. Muscle wasting in the context of T2D and obesity represents not only a complication but also a driver of disease progression, contributing to diminished insulin sensitivity, reduced physical capacity and adverse outcomes [3]. Indeed, sarcopenia has been associated with impaired mobility, increased frailty, higher hospitalization rates and excess mortality [4]. In recent years, glucagon-like peptide-1 receptor agonists (GLP-1RAs) have emerged as cornerstone therapies in the management of T2D and obesity [5–7]. Their multifaceted benefits, including significant improvements in glycemic control and weight reduction, together with cardiovascular and renal protection, have led to their widespread use across various clinical settings [8–10]. However, weight loss induced by GLP-1RAs encompasses both fat and lean tissue compartments and could be associated to nutritional deficiencies, raising important questions regarding their impact on skeletal muscle, particularly in individuals at increased risk of sarcopenia [11]. Despite variability across studies, body composition analyses from clinical trials have shown that a relevant proportion of total weight reduction under GLP-1RAs therapy can be attributed to the loss of lean mass, including skeletal muscle [12]. Whether such changes represent a physiological adaptation to overall weight reduction or pose a risk for functional decline remains an unresolved issue [13, 14]. Moreover, the clinical significance of lean mass loss cannot be inferred solely from quantitative measures, as muscle quality, strength, and metabolic function may be preserved or even improved in some contexts [15–17]. These uncertainties underscore the need for a comprehensive evaluation of the interplay between GLP-1RAs therapy and skeletal muscle dynamics. The present review aims to fill this gap of knowledge by evaluating the existing evidence on muscle wasting associated with GLP-1RAs use and by exploring prevention strategies and treatment approaches to maintain muscle mass and function during GLP-1RAs therapy.

## Epidemiology

The prevalence of sarcopenia among individuals with T2D ranges from 7% to 29%, depending on the diagnostic criteria and study population, with higher rates observed in older adults and in patients with long-standing disease [1]. In people with obesity, particularly in cases of rapid or marked weight reduction, the decline in lean mass may account up to 25–30% of the total weight loss, contributing to the so-called “sarcopenic obesity” [18]. It is important, however, to differentiate between the physiological loss of lean mass that naturally accompanies fat mass reduction and the development of clinically meaningful sarcopenia. The former reflects a proportional decrease in lean tissue during negative energy balance and is typically expected to account for roughly one-quarter of the total weight loss, whereas the latter involves both quantitative loss of skeletal muscle and impaired muscle function or strength [19]. Loss of lean tissue, comprising skeletal muscle, bone, organs, and body water, is therefore an important consideration in patients undergoing weight reduction with GLP-1RAs. Unlike sarcopenia, which requires both loss of muscle quantity and reduced function, lean mass reduction is a body-composition outcome most often assessed using dual-energy X-ray absorptiometry (DXA) or bioimpedance analysis (BIA) [2, 20]. Clinical trial evidence consistently shows that GLP-1RA therapy results in significant reductions in fat mass, accompanied by smaller but measurable decreases in lean mass. In the STEP 1 trial, semaglutide produced substantial weight loss, of which approximately 30% was attributed to lean tissue, while fat loss predominated [21, 22]. Similar findings were reported in analyses of tirzepatide, where about three-quarters of the weight loss was fat mass and one-quarter was lean mass, proportions that closely resemble those observed with diet-induced weight loss [14, 23]. Observational data suggest that muscle wasting is not disproportionately accelerated by GLP-1RAs compared to other weight-loss interventions. A cross-sectional study of older adults treated with semaglutide reported a prevalence of reduced muscle mass of 27.7%, reflecting the high baseline risk in this population rather than drug-specific effects [24]. Systematic reviews and meta-analyses of randomized trials have further confirmed that while GLP-1RAs lower body weight and fat mass significantly, they do not appear to exert an independent detrimental effect on skeletal muscle index beyond expected proportional reductions [25]. Importantly, physical performance outcomes such as mobility and functional capacity are generally maintained, indicating that moderate lean mass loss during therapy does not necessarily equate to clinically relevant impairment [19, 26]. Despite these insights, the incidence of clinically significant muscle wasting attributable to GLP-1RA therapy remains undefined. Trials to date have focused on weight and metabolic outcomes, with lean mass reduction typically reported as a secondary endpoint and without standardized functional measures. Notably, most available studies lack direct assessments of muscle strength, physical performance, or gait speed. The absence of these endpoints limits the ability to determine whether observed changes in lean mass translate into clinically meaningful impairments. Risk factors for greater lean tissue loss likely include advanced age, rapid or large absolute weight loss, low dietary protein intake, sedentary lifestyle, and comorbid conditions such as chronic kidney disease or long-standing diabetes [15]. Overall, current epidemiologic evidence shows that GLP-1RAs consistently induce fat loss accompanied by proportional lean tissue reduction (Table 1). While lean mass typically accounts for about one-quarter of total weight lost, this pattern is comparable to other weight-loss approaches and not uniquely harmful. Accordingly, moderate lean tissue decline observed during GLP-1RA-induced weight loss could be interpreted as a physiological component of body composition change rather than evidence of pathological sarcopenia, unless accompanied by functional deterioration. However, the incidence of clinically meaningful muscle wasting under GLP-1RA therapy remains to be determined through prospective studies with standardized assessment methods.

**Table 1:** Main clinical trials assessing lean mass change during GLP-1 receptor agonists treatment

Clinical Trial	Drug and study duration	Population	Method for lean mass evaluation	Mean Δ body weight	Mean Δ lean mass	Δ muscle quality/function	References
STEP-1 (DXA substudy)	Semaglutide 2.4 mg wkly; 68 wks	Adults with overweight/ obesity (DXA substudy *n* = 140)	DXA	−14.9%; ~ 34% lean mass	−9.7%; but increased proportion of lean body mass relative to total body mass with semaglutide	No consistent clinically meaningful declines in function	[21, 22]
SUSTAIN-8 (DXA substudy)	Semaglutide 1.0 mg wkly; 52 wks	Adults with T2D (DXA substudy *n* = 178)	DXA	−6%; ~ 43% lean mass (calc)	−4.5% (calc)	No clinically relevant function loss	[26]
SURMOUNT-1 (DXA substudy)	Tirzepatide 5/10/15 mg wkly; 72 wks	Adults with overweight/obesity (DXA substudy *n* = 160)	DXA	−21.3% (pooled doses); ~ 25% lean mass	−10.9%	No signal of clinically meaningful functional decline; higher fat loss fraction	[23]
SURPASS-3 MRI post-hoc (muscle composition study)	Tirzepatide 5/10/15 mg wkly; 52 wks	Adults with T2D (MRI substudy *n* = 246)	MRI	−10.4% (pooled doses)	NA; decrease in muscle volume proportional to weight loss; maintenance of fat-free muscle volume and improved muscle quality (less fat infiltration)	Improvements in muscle quality metrics	[17]

Ref: reference; DXA: dual-energy X-ray absorptiometry, wkly: weekly; wks: weeks; T2D: type 2 diabetes; calc: value calculated considering reported mean baseline and mean absolute change; MRI: magnetic resonance imaging

## Mechanisms of muscle loss associated with GLP-1RAs treatment

**Fig. 1:**
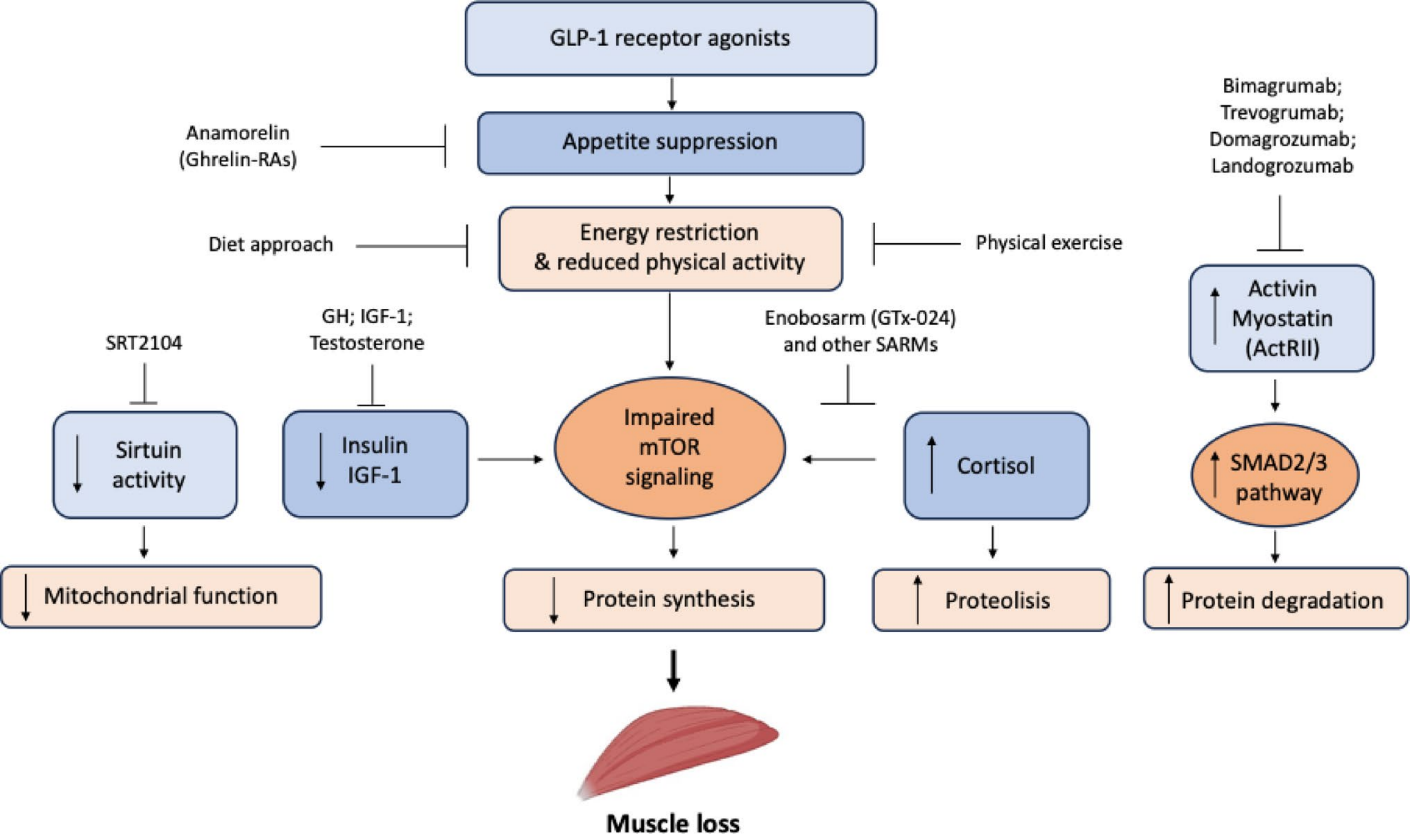
Principal mechanisms of muscles loss associated with GLP-1 receptor agonists treatment and strategies to counterbalance this phenomenon. GLP-1: glucagon-like peptide-1, RAs: receptor agonists; GH: Growth Hormone, IGF-1: insulin-like growth factor-1, mTOR: mammalian target of rapamycin, ActRII: activin type II receptors, SMAD2/3: small mother against decapentaplegic 2/3, SARMs: selective androgen receptor modulators

Preservation of skeletal muscle is critical for metabolic health, physical function and long-term outcomes, making the study of mechanisms underlying muscle loss during GLP-1RA therapy clinically relevant. The primary mechanism through which GLP-1RAs induce weight loss is appetite suppression and delayed gastric emptying, mediated via hypothalamic and brainstem pathways [27]. The reduction in adiposity may help protect muscle tissue from muscle wasting, which is often seen in obesity-associated catabolic states [28], in addition, sustained negative energy balance promotes fat loss, but also contributes to lean tissue catabolism. Approximately 25% of total weight loss typically originates from fat-free mass, with skeletal muscle comprising about half of this component [29]. Reduced protein intake, a common consequence of appetite suppression, particularly in older adults or those with low baseline muscle mass, further predisposes to loss of lean tissue [30]. Hormonal modulation is involved in the anabolic and catabolic processes as well. Insulin and insulin-like growth factor-1 (IGF-1) promote anabolic signaling in skeletal muscle primarily via the PI3K-Akt-mTOR pathway. GLP-1RAs enhance insulin sensitivity and lower fasting insulin levels. However, under caloric restriction, these changes can paradoxically reduce anabolic signaling, impairing protein synthesis. Decreased insulin and IGF-1 signaling lowers mTOR activity, shifting the balance toward muscle catabolism [31]. Negative energy balance and weight loss can activate the hypothalamic-pituitary-adrenal axis, elevating cortisol levels. Increased cortisol stimulates muscle proteolysis through the ubiquitin-proteasome pathway and antagonizes insulin’s anabolic effects, further contributing to lean mass loss [32]. GLP-1RAs may also influence growth hormone (GH) secretion, which is integral to maintaining lean mass via IGF-1-mediated protein synthesis. Modulation of GH secretion may enhance anabolic processes in liver and muscle, partially counteracting catabolic effects [33]. The mammalian target of rapamycin (mTOR) integrates nutrient availability, energy status and growth factor signals to regulate protein synthesis. GLP-1RA-induced reductions in protein intake, lower insulin/IGF-1 and negative energy balance converge to suppress mTOR signaling, reducing muscle protein synthesis [31]. While GLP-1RAs can directly stimulate mTORC1 in some contexts, the net effect of the impaired mTOR signaling during sustained caloric deficit may favor catabolism [34]. Weight loss and caloric restriction increase muscle proteolysis, providing amino acids for gluconeogenesis and acute-phase protein synthesis. GLP-1RAs may modulate inflammation by reducing circulating pro-inflammatory cytokines such as TNF-α and IL-6, which promote proteolysis via the ubiquitin-proteasome pathway [9]. This anti-inflammatory effect may partially counterbalance catabolic stimuli, though lean mass loss remains a concern. Skeletal muscle mitochondrial function is essential for muscle quality and metabolic capacity. Animal studies suggest that GLP-1 signaling can influence mitochondrial dynamics and oxidative phosphorylation. Improved mitochondrial efficiency has been observed, but prolonged caloric restriction may reduce mitochondrial biogenesis and oxidative capacity, compromising muscle function even if absolute mass loss is modest [35]. Older adults and individuals with pre-existing sarcopenia are particularly susceptible to lean mass loss. Age-related anabolic resistance diminishes the muscle-preserving effects of protein intake and exercise during weight loss [36]. Observational studies indicate that older GLP-1RA users experience greater proportional lean mass loss than younger adults [14]. Although GLP-1RAs improve mobility and reduce body weight, some patients decrease physical activity during treatment due to fatigue or altered energy perception. Reduced mechanical loading decreases the stimulus for muscle protein synthesis, exacerbating lean tissue loss [31]. Altogether, muscle loss during GLP1RAs therapy is multifactorial, involving reduced protein intake and negative energy balance, hormonal adaptations, impaired mTOR signaling and protein synthesis, increased proteolysis and potential mitochondrial adaptations, and age, frailty, and physical inactivity as modifiers (Fig. 1). Further research is however needed to elucidate the long-term impact of GLP-1RAs on skeletal muscle and to optimize interventions that preserve muscle mass while retaining metabolic benefits.

## Prevention of muscle loss with dietary approach

Given the sarcopenia-related increase in morbidity and mortality, preventing muscle mass loss in patients taking weight-loss drugs, such as GLP-1RAs, is of primary importance and should include both physical exercise and dietary strategies [37]. Observational real-world data in patients with T2D receiving oral semaglutide for 26 weeks demonstrated preservation of fat-free and skeletal muscle mass despite significant losses in fat mass and visceral adipose tissue, suggesting that the magnitude of lean tissue change may depend on concomitant lifestyle behaviors, such as physical activity and diet [16]. Tailored resistance exercise training can optimize changes in body composition by preserving lean mass while achieving fat loss when associated to hypocaloric diet or incretin therapy [38, 39]. Besides physical activity, diet constitutes a key element in the prevention of muscle loss. In particular, adequate protein intake represents the cornerstone of nutritional prevention [40]. Hypocaloric diets, when not adjusted for protein density, increase the risk of disproportionate lean mass loss [41]. Based on current evidence, several practical recommendations can be outlined. According to a joint Advisory from the American College of Lifestyle Medicine, the American Society for Nutrition, the Obesity Medicine Association, and The Obesity Society, a daily protein intake of 1.2–1.6 g/kg adjusted body weight, should be considered in individuals initiating GLP-1RA therapy, particularly when caloric restriction is prescribed. Protein intake in adults should not fall below 0.4–0.5 g/kg/day to avoid muscle atrophy and functional impairments, whereas prolonged intake at or above 2 g/kg/day should be avoided due to potential adverse health effects [42]. However, in a recent cross-sectional analysis of GLP-1RAs users, only 43% of participants achieved the minimum recommended intake of 1.2 g/kg/day, with < 10% reaching higher targets such as 1.6–2.0 g/kg/day, and with an average protein consumption accounting for just 18–19% of daily energy intake [43]. This gap highlights the clinical need for targeted nutritional counseling and, when necessary, supplementation. Moreover, periodic body composition assessment (e.g., through DXA or BIA) is advisable in order to early detect disproportionate loss of lean mass and to adapt nutritional interventions accordingly [42]. The relevance of protein intake lies not only in the total daily amount, but also in its distribution across meals. Skeletal muscle exhibits a per-meal threshold for protein synthesis, with approximately 20–25 g of high-quality proteins being sufficient to maximize the postprandial anabolic response in adults. Proteins consumed beyond this threshold do not further enhance muscle protein synthesis due to the so-called ‘muscle-full’ effect, with the excess amino acids preferentially oxidized [44]. Several studies have also suggested that a more uniform distribution of protein across meals is associated with superior anabolic outcomes compared to a skewed pattern. Trials in young and middle-aged adults have shown higher rates of overall protein synthesis when ~ 30 g of protein were consumed in each of the three meals [45]. Similarly, observational data from the NuAge study in older Quebec adults linked a more balanced protein distribution with greater appendicular lean mass [46]. Nonetheless, clinical evidence remains inconclusive. Indeed, an opposite approach, as the “pulse feeding” strategy, whereby ~ 80% of daily protein is consumed in a single meal, has been shown to improve nitrogen retention in older women and to enhance lean mass preservation in malnourished elderly patients [47]. In addition, protein ingestion in close temporal proximity to resistance exercise appears to further potentiate hypertrophic adaptations, particularly in older adults. In a study by Esmarck et al., immediate post-exercise protein supplementation during a 12-week resistance training program in old men, induced significant quadriceps hypertrophy, whereas delayed supplementation had no effect [48]. Taken together, these findings suggest that balanced protein distribution across meals and strategic timing of intake around resistance exercise may enhance muscle mass preservation, however, the optimal feeding pattern is likely context-dependent and influenced by age, nutritional status and comorbidities. Beyond protein quantity, the source of protein also plays a key role in metabolic regulation. Whey proteins, rich in leucine, and plant-based diets have been shown to support fat free mass preservation in individuals with obesity. Moreover, specific amino acids, like branched chain amino acids (BCAA), methionine, glutamate, tryptophan and its metabolites, can beneficially influence obesity-related parameters and complications [49]. Supplements such as BCAA (5–12 g/day), leucine (6 g/day), β-Hydroxy-β-methylbutyrate (3 g/day), creatine (20 g/day for 5–7 days, followed by maintenance dose of 3–5 g/day), omega-3 fatty acids (2–4 g/day) and vitamin D (800–1000 IU/day) may offer additional benefits in improving the health and quality of life of patients with sarcopenia, especially when balanced hypocaloric diet with adequate protein intake and physical exercise are insufficient to maintain muscle mass and function [50]. In obese older adults undergoing a hypocaloric diet combined with resistance training, supplementation with whey proteins, leucine, and vitamin D, associated to a protein intake of ~ 1.1 g/kg vs. 0.85 g/kg in the isocaloric control group, preserved appendicular muscle mass during weight loss compared to the control condition [51]. The leucine metabolite, β-Hydroxy-β-methylbutyrate has been shown to promote anabolism and limit muscle catabolism [52, 53]. Preliminary data suggest that β-Hydroxy-β-methylbutyrate may attenuate lean mass loss during caloric restriction [54]. Creatine supplementation, when combined with resistance training, may increase lean mass and muscle strength. Nevertheless, evidence regarding its effects on muscle mass in the absence of exercise remains inconclusive [50]. n-3 polyunsaturated fatty acids from fish oil exert anti-inflammatory and anabolic effects on skeletal muscle. In older adults with stable weight, supplementation with 3–4 g/day of eicosapentaenoic and docosahexaenoic acids for six months improved handgrip strength and lean mass [55]. Vitamin D is known to contribute to muscle and bone health [56]. A meta-analysis reported a modest positive effect of vitamin D supplementation on muscle strength in elderly individuals with deficiency, but no effect on muscle mass, nor on muscle preservation during caloric restriction [57, 58]. Finally, other antioxidant vitamins, like vitamin C and E, and anti-inflammatory phytonutrients, as curcumin and polyphenols, may support muscle recovery and mitigate exercise- and diet-induced oxidative stress, although further studies are needed to determine their direct impact on lean body mass during energy restriction [59–61]. Collectively, emerging evidence suggests that the most effective approach to prevent muscle wasting during GLP-1RA therapy combines nutritional and exercise strategies, with a multidisciplinary clinical management involving physicians, dietitians and exercise specialists to tailor interventions based on individual goals, comorbidities and age. Key components include resistance training and daily moderate aerobic exercise, adequate high-quality protein intake (1.2–1.6 g/kg adjusted body weight/day) distributed evenly across meals, micronutrient supplementation, and periodic reassessment of lean mass via DXA, BIA or functional outcomes (e.g., handgrip strength and gait speed) to optimize fat loss while minimizing sarcopenia risk (Table 2). Although definitive randomized trials specifically designed to test proteins, amino acids and micronutrients supplementation in this population are still lacking, data from clinical trials, real-world studies, and cross-sectional analyses support the adoption of a preventive and structured nutritional framework in clinical practice.

**Table 2:** Strategies to prevent muscle loss during GLP-1 receptor agonists treatment

Strategy	Intervention	Mechanism/Rationale	Evidence
Physical Activity	Resistance training	Preserves lean mass	[38, 39]
	Moderate aerobic exercise	Complements resistance training	
Nutrition	Adequate protein intake (1.2–1.6 g/kg/day) and timing	Supports muscle protein synthesis	[42, 44–48]
	Amino acid supplementation (BCAA, leucine-enriched)	Activates mTOR; stimulates protein synthesis	[49–54]
	Omega-3 fatty acids and Micronutrients (vitamin D, C, E, B12, iron, ect.) supplementation	Prevents deficiencies	[50, 51, 55–61]
Medical Supervision	Body composition monitoring (DXA, BIA)	Detects early muscle loss	[42]
	Personalized GLP-1RAs dosing	Tailors diet and exercise	

GLP-1RAs: glucagon-like peptide-1 receptor agonists, BCAA: branched chain amino acids, mTOR: mammalian target of rapamycin, DXA: dual-energy X-ray absorptiometry, BIA: bioimpedance analysis

## Novel pharmacological approaches

Besides behavioral strategies, emerging pharmacologic agents, comprising anabolic and anti-catabolic drugs, have been demonstrated to be effective in the prevention and treatment of sarcopenia or disproportionate lean mass loss in patients receiving GLP-1RAs. Activin type II receptor (ActRII) blockade is the most advanced mechanistic approach. Bimagrumab, a monoclonal antibody targeting ActRIIA/IIB, inhibits myostatin and activins, promoting muscle hypertrophy and reducing fat mass. Early phase II studies in older adults with sarcopenia showed increases in lean mass and, in slower walkers, improvements in gait speed and 6-minute walk distance [62]. A larger randomized study found significant gains in lean mass versus placebo but no clear overall advantage in physical performance when both groups received optimized nutrition and exercise [63]. In adults with type 2 diabetes and obesity, a 48-week phase II trial demonstrated increased lean mass, reduced fat mass and glycemic improvement [64]. Meta-analyses confirm robust effects on body composition, but variable functional outcomes, highlighting the importance of patient selection and concomitant exercise/nutritional strategies [65]. Given its dual effect on muscle and fat, ActRII blockade is an attractive adjunct to GLP-1RAs to mitigate lean mass loss. Combination trials with GLP-1/GIP agents are in progress, although functional endpoints and long-term safety remain insufficiently defined. Emerging multi-agonists such as retatrutide (GLP-1/GIP/glucagon triple agonist) and PTT-A (novel unimolecular peptide tetra-agonist targeting GLP-1, GIP, glucagon and amylin receptors) further expand this concept. Retatrutide appeared to induce lean mass loss in similar proportions to GLP-1RAs alone, whereas PTT-A has shown robust fat reduction without significant decrease in muscle mass, highlighting the potential of selective multi-agonists to preserve skeletal muscle during weight loss [66, 67]. Other inhibitors of the myostatin/activin pathway (e.g. trevogrumab, domagrozumab, landogrozumab) have shown increases in lean mass across different populations, including Duchenne muscular dystrophy, but strength and function results are inconsistent [68]. Similarly, the sirtuin 1 activator, SRT2104, demonstrated to mimic the beneficial effects of exercise and enhance muscle function and regeneration, and mitochondrial function, thereby promoting recovery in Duchenne muscular dystrophy models [69]. Full publication of combination-therapy data is awaited, however interim analysis from the phase II COURAGE trial (NCT06299098) found 3.3% reduction in lean mass of obese patients treated with the combination of trevogrumab and semaglutide, compared with 6.5% reduction with semaglutide alone [70]. Selective androgen receptor modulators (SARMs), such as enobosarm (GTx-024), increase lean mass and may improve function, but phase III results have been mixed and no agent has regulatory approval for sarcopenia [71]. Ghrelin receptor agonists like anamorelin increase lean mass and stimulate appetite in cancer cachexia [72], but appetite stimulation may be counterproductive alongside GLP-1-mediated appetite suppression. Anabolic hormones (e.g. testosterone, growth hormone, IGF-1) can increase lean mass in selected patients, but require specialist oversight and carry safety concerns [73]. At present, no pharmacologic agent is standard of care to preserve muscle during GLP-1RA therapy. ActRII inhibitors such as bimagrumab have the strongest body-composition data, but consistent functional benefits and long-term safety in diverse populations are lacking. Future trials should prioritize sufficient duration (≥ 12–18 months), include high-risk subgroups (e.g., older adults, frail individuals, patients with rapid or marked weight loss, or chronic comorbidities such as chronic kidney disease), and integrate nutritional and resistance training interventions. Key questions encompass timing of intervention (preventive vs. rescue use) and employment of combination strategies with multi-agonist incretin therapies and/or anabolic/anti-catabolic agents. Moreover, functional outcomes, including muscle strength, gait speed and disability falls, should be adopted as primary endpoints rather than secondary measures to ensure meaningful clinical translation. Non-pharmacologic strategies remain the foundation. Resistance training and adequate protein intake should be prioritized, with monitoring of muscle mass and function during GLP-1RA treatment. For high-risk patients or those with documented decline despite optimal lifestyle measures, referral to clinical trials of emerging anabolic/anti-catabolic therapies is appropriate. Industry activity suggests rapid development in this area [14], but clinical adoption will depend on functional benefits, safety, cost-effectiveness and regulatory guidance. Preventing excessive muscle loss during GLP-1RA therapy ultimately requires a multimodal strategy. While anabolic or anti-catabolic agents - especially myostatin/activin pathway inhibitors - show promise, translation into improved patient-centered outcomes is not yet established (Table 3). Continued research and careful outcome selection will be essential for future integration into diabetes and obesity care.

**Table 3:** Pharmacological options to increase lean mass during GLP-1 receptor agonists treatment

Class/mechanism	Example agents	Evidence on lean mass	Functional outcomes	Current status	References
ActRII (myostatin/activin) inhibitors	Bimagrumab; Trevogrumab; Domagrozumab; Landogrozumab	Consistent ↑ lean mass and ↓ fat mass in sarcopenia, T2D, and obesity trials	Mixed; improvement in some subgroups (gait speed, 6MWD)	Not approved; trials ongoing; combinations with GLP-1/GIP agents under investigation	[62– [65, 68, 70]
Myostatin-targeted antibodies (subset)	Domagrozumab; Landogrozumab	↑ Lean mass in DMD and older adults	Limited or inconsistent functional outcomes	Development stage; awaiting larger studies	[68]
Sirtuin 1 activators	SRT2104	↑ Muscle function and regeneration in DMD models	Inconsistent clinical outcomes	Not approved; under investigation for several diseases	[69]
SARMs	Enobosarm (GTx-024) and others	↑ Lean mass in phase II studies	Inconsistent strength/ functional benefits	No approval for sarcopenia; further trials needed	[71]
Ghrelin receptor agonists	Anamorelin	↑ Lean mass and appetite in cancer cachexia	Limited functional endpoints; appetite stimulation may counteract GLP-1RAs induced anorexia	Approved in some regions for cachexia; less synergistic with GLP-1RAs	[72]
Anabolic hormones	Testosterone; Growth Hormone; IGF-1	↑ Lean mass in deficient/hypogonadal patients	Variable effects; safety concerns (CV, prostate, etc.)	Off-label/specialist use; monitoring required	[73]

ActRII: activin type II receptors, T2D: type 2 diabetes, 6 MWD: 6-minute walk distance, GLP-1/GIP: glucagon-like peptide-1/glucose-dependent insulinotropic polypeptide, SARMs: selective androgen receptor modulators, DMD: Duchenne muscular dystrophy, GLP-1RAs: GLP-1 receptor agonists, IGF-1: insulin-like growth factor, CV: cardiovascular

## Conclusions

Skeletal muscle wasting is a frequent and clinically significant complication in patients with T2D and obesity, which is exacerbated by chronic inflammation, insulin resistance and metabolic dysregulation. While highly effective for weight loss and cardiometabolic protection, GLP-1RAs may contribute to lean mass reduction, highlighting the importance of monitoring muscle health during therapy. Integrated strategies combining adequate protein intake, resistance exercise, and, where appropriate, emerging pharmacological interventions can help preserve or restore muscle mass. Further research, including long-term evaluation of sarcopenia in GLP-1RAs trials, development of standardized monitoring protocols and investigation of combined nutritional, exercise and pharmacologic strategies, is needed in order to prevent muscle loss while maximizing metabolic benefits under GLP-1RAs treatment.

## Data Availability

Data sharing is not applicable to this article as no new data were created or analyzed in this study.
